# Data mining and decision support systems for efficient dairy production

**DOI:** 10.14202/vetworld.2021.1258-1262

**Published:** 2021-05-22

**Authors:** Sunesh Balhara, Rishi Pal Singh, A. P. Ruhil

**Affiliations:** 1 ICAR-Central Institute for Research on Buffaloes, Hisar, Haryana, India; 2 Department of Computer Science and Engineering, Guru Jambheshwar University of Science and Technology, Hisar, Haryana, India; 3 ICAR-National Dairy Research Institute, Karnal, Haryana, India

**Keywords:** dairy farming, data mining, decision support systems

## Abstract

Gainful livestock farming requires selective breeding of animals with certain heritable desirable traits which gives profitability in terms of farm produce. Modern dairy animals are selected for traits which directly or indirectly contribute to high milk production. The concept of “feed conversion efficiency” in terms of milk production is now vigorously taken up by researchers and farm managers for recognizing and breeding efficient milk-producing animals. The whole concept of economic farming thus requires identification of “elite” animals, meeting above criteria as base population for the farm enterprise. Farmers and animal traders have been selecting best animals based on certain physical characters, which were also accepted by the breeding scientists as phenotypes. Data mining allows uncovering of hidden patterns in the data for better understanding of data relationship for developing suitable models for further improvements. Along with artificial intelligence techniques, data mining has opened new avenues for achieving high resource utilization efficiency and sustainable profitability in livestock production systems. The present review discusses and summarizes various data mining techniques and decision support systems for scientific dairy farming.

## Introduction

The animal rearing has become highly competitive with narrow profit margins and therefore, there is increased focus on research for improving the efficiency of animal production. Proper selection of animals is the first and the most important step in dairy farming. Animal records, which can be utilized in data mining-based animal evaluation, form the basis of selection for breeding programs and/or a new dairy herd. For this, farmers need to be well informed about best practices in the selection of elite animals. It is not uncommon for the animal breeders to ask: Which is the best dairy animal? When an owner offers an animal for sale, he must be putting on animals that are being culled from the herd for undesirable traits. Therefore, the purchaser has to be well aware of the characters of a good animal.

For any dairy setup, most common criteria for the selection of dairy animals for breeding, production and sale/purchase include –

1. Milk production potential – High milk yield is preferred because of higher economic returns it gives
2. Milk composition – In India, fat content is a key factor in determining price of milk. However, in western countries, fat, protein, and solid not fat are considered. Buffaloes with high milk fat and protein content are preferred for Mozzarella cheese production
3. Fertility – Animals with a pedigree history of regular breeding are desired
4. Sex of the offspring – Female offspring is preferred for dairying while male is preferred for raising bulls for natural service or semen production
5. Performance of the breed in the local environment – Better climate resilient breeds are preferred
6. Resistance against prevalent diseases – Disease resistant animals incur lesser expenses on healthcare and hence preferred over vulnerable animals
7. Age of the animal – Age of the animal determines the longevity of the animal in farm and hence its production life. Therefore, younger animals are preferred
8. Feed conversion efficiency – It is an indicator of how efficiently the animal utilizes feed offered for conversion into animal produce (for lactating animals, it is milk). Thus, animals with higher efficiency will produce more with same amount of feed offered and hence selected
9. True to breed characters – These are indicative of good production potential and high resale value
10. Disposal of spent/unproductive animals – Cow slaughtering is not allowed in India but buffalo slaughtering is permissible, so spent/unproductive buffaloes realize higher price and therefore preferred by many farmers for dairy.

A good farm animal is not universal and much depends on what you want and where you are. Researchers have used data mining tools on recorded data for the development of intelligent decision support systems. The present review provides an insight into the progress made with regard to finding smart computer-based solutions to various problems related to commercial animal farming.

## Quantifying Traits through Selection Indices

As elaborately above, for success of any dairying program, identification of the best animals is a prerequisite. Conventionally, farmers have developed certain assumptions with respect to good dairy characters and this indigenous technical knowledge has been passed on from generation to generation. Animal breeders and dairy managers have added a lot of information on scientific assessment of the animal for their selection. It has now been widely accepted that there is high level of correlation between physical characters (“phenotypes”) and the production potential of the animal. Farmers, animal lovers, researchers, and breeders have been following “Score Card” methods for various type of animals’ selection during judging in animal championships. Scientifically, animals are evaluated in terms of heritability of characters and genetic gain in subsequent generations [1]. Globally, animal breeding programs have identified and quantified heritability of different traits for improvements in production in subsequent generations. These traits are controlled by certain genes, which allow calculations of genetic correlation. These heritability scores have been used for classification of traits in Holstein and Jersey cows in USA [2]. Data collected on such traits along with genetic parameters have paved way for the development of indices, which can be applied at dairy farms for breed improvement programs, for example, “Net Merit Dollars” index used by the United States Department of Agriculture ranks dairy cows in economic terms. Similarly, the selection indices Jersey Performance Index for Jersey and Total Performance Index for Holstein cattle were developed by the American Jersey Cattle Association and Holstein Association, USA, respectively. These breed-specific selection tools are updated periodically to include latest scientific knowledge and economic aspects of dairying.

## Dairy Production Expert Systems Based on Artificial Neural Network (ANN) and Fuzzy Logic

Statistical analysis of the historical animal production data and subsequently its data mining applications gave impetus to for developing smart dairying solutions including automation of operations. ANN and fuzzy logic are most important arithmetic tools that have been widely used in developing artificial intelligence (AI) for decision making machines. Introduced by Lotfi Asker Zadeh in 1965, fuzzy logic (or fuzzy rule) models are based on the principal of logic, which is similar to the human reasoning ability [3]. The earliest efforts for developing AI focused around comprehensive breeding plan for productivity improvement in livestock population. Animal production systems are greatly affected by diseases, and this necessitated development of expert systems for disease diagnosis and surveillance. Animal nutrition management is another area where expert systems are in great demand as this particular activity accounts for the largest portion of recurring costs in livestock farming.

Many studies have indicated higher accuracy of ANN and fuzzy system models for calculating breeding estimates: (1) First lactation 305-day milk yield [4-6], (2) first lactation 305 days or less milk yield (FL305DMY) from early body weights [7], (3) multiple traits – AFC, FL305DMY, first lactation length, first service period, first dry period, etc. [8], and (4) estimated breeding values [9]. Conventionally, the best linear unbiased prediction method has been used for estimating milk and fat yields in dairy cows. A multilayer program has been used to build ANN and neuro-fuzzy systems to predict breeding values in cows [9]. The model, although not user friendly, had higher degree of accuracy over conventional breeding evaluation methods.

Automation in milking at large-sized dairy farms facilitated accumulation of large volumes of accurate data and development of expert systems for its earliest detection of mastitis. Mastitis, affecting dairy production globally, is one of the most extensively studied dairy animal diseases. Early detection of mastitis helps in checking its further spread and minimizing chances of permanent udder losses. A fuzzy logic model for classification and control of mastitis was developed using a data set of 403,537 milking’s from 478 cows [10]. Mastitis was determined according to somatic cell counts (SCC) standards. Mastitis alerts were generated by a fuzzy logic model using MATLAB software with electrical conductivity, milk production rate and milk flow rate as input data. After evaluation on specific parameters, the model was proposed as a useful tool for the development of an intelligent mastitis detection method. In another study, ANN and adaptive neuro-fuzzy inference system models were developed for raising mastitis alerts (subclinical stage) on the basis of SCC in Holstein cows [11]. A cost-effective neural network (NN) model using combination of variables – pH, electrical conductivity, temperature (udder, milk and skin), milk somatic cells, milk yield, and dielectric constant, was developed to classify healthy and mastitis Murrah buffaloes [12]. A fuzzy logic based automated monitoring system for sorting mastitis and estrus alerts in cows [13]. The output was in the form of “true” or “false,” for each alert. The number of false-positive alerts did not change the level of detected cases of mastitis and estrus. Lameness is another gray area in dairy production, which requires immediate animal replacements in the herd. A very basic fuzzy model to predict lameness was developed for cows using correlation between claw length and dietary intake in cows [14]. Since the model was very basic, it was not of much use. More sophisticated, inclusive and accurate lameness detection systems have now come up which can alert up to three days in advance of manual observation [15].

Data science is of enormous importance in poultry production. A neuro-fuzzy algorithm – combination of ANN and fuzzy logic, for poultry feed formulation is a useful tool wisely used in African poultry industry [16]. The algorithm in this model was trained with African feed ingredients composition on MATLAB 2009 platform. Outputs from the system were compared with some available standards and the data analyzed on NCSS 2000. The system produced high accuracy of results and fulfills the objectives of reducing feed cost and supplementing all essential minerals and amino acids in poultry diet – Arginine, Histidine, Isoleucine, Leucine, Lysine, Phenylalanine, Tyrosine, Cystine, Methionine, Threonine, Tryptophan, Valine; Crude protein; Ether Extract; and Crude Fiber.

## Decision Support Systems (DSS) in Livestock Production

The decision support systems help a setup (organization, individual, etc.) in decision making processes by the use of computer-based models to identify and solve problems [17]. DSS essentially work on the principal of knowledge acquisition from current or historical data followed by creating appropriate algorithm for logical decision-making for identifying and solving problems in the system. The decision support systems are being recognized as tools for improving resource utilization across businesses and other enterprises. For livestock, mostly “data-driven” or “knowledge-driven” DSS are in use or developed, as these are based on analysis of large amounts of structured data/information [18].

During the last decade of twentieth century, a very basic DSS was developed for pasture-based livestock production [19]. This model was a good effort in simulating biological processes (grass growth, pasture-animal interface, and cow model), population dynamics (stochastic herd model), management practices, and economics for suggesting options on efficient use of resources for optimum farm production.

With accumulation of scientific data over the years, better understanding of animal biology and recent developments in data mining applications, DSS for specific purposes are now in use in livestock production. Most DSS have been developed from simulation models using multiple computational methods.

Nevertheless, these systems offer great benefit to the dairy enterprises by assisting decision-making process in the management programs. Table-1 [20-27] lists some of the commercially available DSS which have been well adopted by farmers and frequently updated incorporating scientific breakthroughs.

**Table-1:** Some common commercially available DSS in dairy farming.

Name of DSS	Application	DM/AI techniques	Reference
GrazPlan suite – GrazFeed, GrazGro and AusFarm	For farmers to make decisions about farm management principally in grazing enterprises	Simulation model	[20]
EUEDE	Assist rural business stakeholders in building their own target-relevant decision-making tools in Australia	Generic knowledge model	[21]
VetMet	Surveillance and control of airborne animal diseases	Linearized flow model	[22]
Farmax Dairy Pro	A pastoral grazing model of a dairy farm	Whole-farm system models	[23]
DairyMGT suite - UW-Dairy Repro$	A collection of tools for decision support systems in Dairy Farm Management	Matrix-based	[24]
ValorE	Livestock manure management	ANN	[25]
ADSDSS	For assisting control of animal diseases – provide information about infected animals, infected places and diseases outbreaks	Apriori algorithm	[26]
Sustainable Organic and Low-input Dairying (SOLID)	To optimize the management of feed resources and feed supply systems within organic and low input dairy systems in Europe to minimize the risk of feed shortages	Simulation model	[27]

ANN=Artificial neural network, EUEDE=End user enabled design environment, ADSDSS=Animal Diseases Spatial Decision Support System

Efforts to develop an expert system for replacing human expert in dairy cattle judging (based on 12 traits – chest width, fore udder attachment, loin, suspensory ligament, angularity, udder depth, rear leg side view, front teat placement, rear leg rear view, rear teat placement, foot angle, and body depth) by linear approach using data mining applications has been tried in Iran [28]. Judging rules were represented as knowledge and fuzzy logic arithmetic was developed to overcome uncertainty during automatic judging. A similar system was developed as a fuzzy logic-based decision support system using reproduction parameters and milk yield records of Holstein Friesians [29] - 305-day milk yield (305 DMY), service period (SP), calving interval (CI), artificial insemination (AI), and dry period (DP) as input parameters. Output parameter of the system is defined as classification decision. Kappa statistics were used to investigate the similarities between expert and decision system and fitting value was obtained as 92.6% (p<0.05).

Computational intelligence techniques have also been used for development of a decision support system in dairy cattle health management [30]. Here, ANNs were used to classify health of cattle into three classes (regular, surveillance, and risk) based on the quality of milk produced. DSS has made way into the concept of precision farming. The Long Short-Term Memory (LSTM) NN based DSS [31] predict forage mass using historical pasture growth data collected directly and association with meteorological data. The results show that LSTM NNs are capable of making a reasonable estimate of the dry mass variation over time. A unique buffalo behavior decision support system, based on threshold value of various acoustic features of vocalization for estrus detection was developed using machine learning techniques J48 classifier along with C4.5 Algorithm to address the problem of silent estrus in buffaloes [32].

## Conclusion

Sustainable dairy farming requires comprehensive decisions in husbandry practices for efficient utilization of resources, better health, and productivity. The software programming should enable us to manage a database consisting of every animal on the farm and utilize the database using data mining techniques and decision support systems. Advances in arithmetical systems have given rise to modeling farm situations close to real and therefore provide highly efficient DSS framework to the stakeholders. The tech-savvy dairy farmers are investing in computer-based tools and software products to improve farm economics through monitoring farm products quality and quantity, breeding management, feed – fodder and pasture availability, disease control, and prevention and environmental parameters in their surroundings. AI acquired through data mining techniques, decision support systems and algorithms are bound to make smart decisions for efficient and sustainable dairy production systems.

## Authors’ Contributions

SB: Did all the literature search and manuscript writing. RPS and APR: Read the manuscript and made corrections. All authors read and approved the final manuscript.
